# Telemedicine and In-Person Visit Modality Mix and Electronic Health Record Use in Primary Care

**DOI:** 10.1001/jamanetworkopen.2024.8060

**Published:** 2024-04-24

**Authors:** Nate C. Apathy, Garrett Zabala, Kylie Gomes, Patti Spaar, Seth A. Krevat, Raj M. Ratwani

**Affiliations:** 1 National Center for Human Factors in Healthcare, MedStar Health Research Institute, Washington, DC; 2 Department of Emergency Medicine, Georgetown University School of Medicine, Washington, DC; 3 Department of General Internal Medicine, Georgetown University School of Medicine, Washington, DC

## Abstract

This cross-sectional study investigates the association between day-to-day changes in telemedicine share and clinician time spent on electronic health record (EHR) use.

## Introduction

Telemedicine use increased substantially during and after the COVID-19 pandemic^1^ and has the potential to provide low-acuity medical services at lower costs.^2^ However, telemedicine also levies new costs on clinicians.^3^ Telemedicine requires shifting care delivery workflows, as it rarely includes clinical support staff but can involve levels of patient complexity similar to in-person visits.^4,5^ This may increase administrative and electronic health record (EHR) burden for clinicians and increase cognitive costs as clinicians switch modalities. In a recent study,^6^ greater weekly telemedicine visit share was associated with increased EHR time, including after-hours time, mostly spent in documentation. Our study aimed to address 2 gaps: first, whether day-to-day changes in telemedicine share demonstrate a similar association with EHR time; and second, what changes occur in domains of EHR use not examined in previous studies (eg, medical record review, orders).

## Methods

This cross-sectional study combined visit modality data with EHR active use data capturing time spent by primary care physicians (PCPs) in the Cerner EHR system from December 2021 through June 2023 at MedStar Health, a large multispecialty health system in the mid-Atlantic region. We calculated PCPs’ daily telemedicine share as the percentage of the day’s visits conducted via telemedicine and categorized this variable into 5 levels. Because we used deidentified data, this study was deemed exempt and not human participant research by the Georgetown University–MedStar Health Institutional Review Board; we followed the STROBE reporting guideline.

Telemedicine visits were identified via registration and scheduling records. We analyzed 5 measures of active EHR time for each PCP-day: total EHR time, documentation time, medical record review time, order time, and next-day documentation time (only for PCP-days with a consecutive qualifying PCP-day). We calculated descriptive statistics and ran ordinary least squares linear regression models, adjusting for visit volume and physician and calendar-day fixed effects. These models estimate the marginal within-clinician association between each telemedicine share level and our outcomes relative to zero-telemedicine days while adjusting for common temporal trends. We used R statistical software, version 3.6.3 (R Project for Statistical Computing) (tidyverse, fixest packages) for analyses, using 2-tailed hypothesis tests (α = .05).

## Results

The study included 316 PCPs observed across 67 894 PCP-day observations distributed across 5 daily telemedicine share categories (zero daily telemedicine share, 44.7% of all PCP-days; ≤10% share, 17.2%; 11%-25% share, 24.8%; 26%-99% share, 11.1%; and 100% share, 2.2%); mean (SD) overall visit volume, 13.9 (7.2) visits/d (Table). All outcomes demonstrated statistically significant differences across telemedicine share levels. The mean (SD) documentation time for PCPs was 71.3 (54.3) minutes on zero-telemedicine days and 87.1 (50.0) minutes on days with up to 10% telemedicine visits. In regression analyses, days with a mix of visit modalities were associated with significantly greater time for EHR, documentation, and medical record review (Figure). Compared with zero-telemedicine days, 26% to 99% telemedicine days were associated with 14.8 (95% CI, 7.6-22.0) more minutes of active EHR time (5.6% increase, *P* < .001), 4.7 (95% CI, 1.2-8.3) additional documentation minutes (6.0% increase, *P* = .01), and 5.5 (95% CI, 2.8-8.2) additional medical record review minutes (6.2% increase, *P* < .001). Telemedicine share resulted in a negligible increase in order time and had no association with next-day documentation time (Figure).

**Table.** Results for All Outcomes Stratified by Daily Telemedicine Share^a^

Outcome	Findings by daily telemedicine share, mean (SD), min/d				
	Zero	≤10%	11%-25%	26%-99%	100%
PCP-days, No. (%)^b^	29 782 (43.9)	11 889 (17.5)	17 109 (25.2)	7653 (11.3)	1461 (2.2)
Active electronic health record time	231.8 (135.6)	303.4 (121.4)	291.0 (118.2)	278.6 (108.9)	120.0 (99.1)
Documentation time	71.3 (54.3)	87.1 (50.0)	85.8 (48.9)	85.6 (47.8)	40.6 (41.8)
Medical record review time	78.8 (49.3)	105.3 (44.6)	99.3 (44.0)	92.1 (39.5)	39.8 (32.5)
Order time	28.7 (21.6)	38.1 (21.2)	35.4 (20.5)	31.6 (18.9)	12.1 (13.6)
Next-day documentation time	68.5 (52.8)	80.6 (49.4)	80.6 (48.5)	79.4 (47.3)	49.6 (55.2)
Total visit count, No./d	13.9 (9.0)	15.7 (4.8)	13.5 (5.3)	13.0 (5.5)	8.6 (4.3)

Abbreviation: PCP, primary care physician.

^a^ The table shows descriptive statistics of outcomes and model covariates across daily telemedicine share categories. All bivariate differences are significant at the *P* < .001 level.

^b^ Our sample included PCP-days with at least 4 patient visits.

**Figure.**
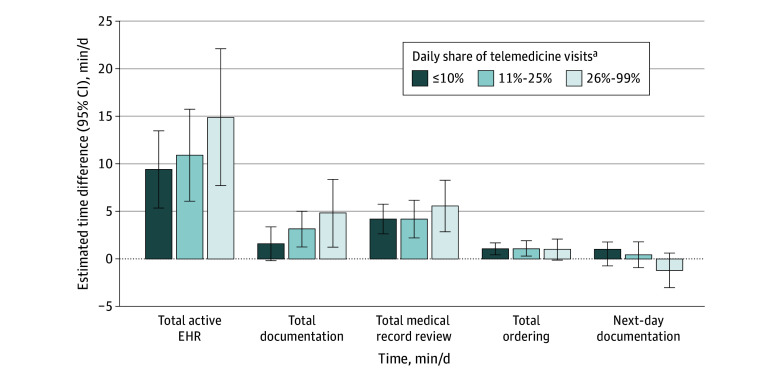
Daily Telemedicine Visit Share and Electronic Health Record (EHR) Time Outcomes The plots show estimates and 95% CIs from ordinary least squares linear regression models adjusting for daily visit volume, and physician and calendar day fixed effects. N = 67 894 for all outcomes except next-day documentation time (n = 47 297). Estimates and 95% CIs for 100% telemedicine days do not reach statistical significance and exhibit wide variation due to relatively few observations; thus, they were omitted for readability. ^a^Reference category is zero telemedicine visits.

## Discussion

This cross-sectional study found that, during clinic days with both telemedicine and in-person visits, PCPs had 5.6% to 6.2% more EHR-based work. This work did not spill over into next-day documentation, suggesting that PCPs absorbed added time into their workload on mixed-modality days. However, we found that fully telemedicine days were not associated with EHR-based work, contrary to previous findings.^6^ We attribute this difference to the small sample of fully telemedicine PCP-days in our study in comparison with prior work^6^ (2.2% vs 16.5% of physician-weeks) as well as higher mean visit volume (13.9 visits/d vs 20 visits/wk) (Table). Greater EHR time may be due to increased multitasking during telemedicine visits, as PCPs simultaneously engage with patients and the EHR during telemedicine visits in ways that are not possible in person. This multitasking may feel more efficient and therefore may not register as “burdensome”; further research should explore whether added EHR time associated with mixed-modality days further burdens PCPs. Limitations of our study include our setting of a single health system, lack of information on visit and patient characteristics and on clinicians’ experience with telehealth tools, and lack of clinical outcomes.

## Notes

Supplement.
Data Sharing Statement

## References

[1] Patel SY, Mehrotra A, Huskamp HA, Uscher-Pines L, Ganguli I, Barnett ML. Trends in outpatient care delivery and telemedicine during the COVID-19 pandemic in the US. JAMA Intern Med. 2021;181(3):388-391. doi:10.1001/jamainternmed.2020.5928 33196765 PMC7670397

[2] Khoong EC. Policy considerations to ensure telemedicine equity. Health Aff (Millwood). 2022;41(5):643-646. doi:10.1377/hlthaff.2022.00300 35500190

[3] Tang M, Chernew ME, Mehrotra A. How emerging telehealth models challenge policymaking. Milbank Q. 2022;100(3):650-672. doi:10.1111/1468-0009.12584 36169169 PMC9576237

[4] Zhong A, Amat MJ, Anderson TS, . Completion of recommended tests and referrals in telehealth vs in-person visits. JAMA Netw Open. 2023;6(11):e2343417. doi:10.1001/jamanetworkopen.2023.43417 37966837 PMC10652149

[5] Chen K, Zhang C, Jackson HB. Relative billing complexity of in-person versus telehealth outpatient encounters. J Eval Clin Pract. 2023;29(6):887-892. doi:10.1111/jep.13905 37515392

[6] Jay Holmgren A, Thombley R, Sinsky CA, Adler-Milstein J. Changes in physician electronic health record use with the expansion of telemedicine. JAMA Intern Med. 2023;183(12):1357-1365. doi:10.1001/jamainternmed.2023.5738 37902737 PMC10616769

