# Supplementary material for: Telemedicine and In-Person Visit Modality Mix and Electronic Health Record Use in Primary Care
Source: JAMA Netw Open. 2024 Apr 24;7(4):e248060. doi: 10.1001/jamanetworkopen.2024.8060 (PMC11043894; doi:10.1001/jamanetworkopen.2024.8060)
Supplement: Supplement. — Data Sharing Statement [file jamanetwopen-e248060-s001.pdf]

Data Sharing Statement

Apathy. Telemedicine and In-Person Visit Modality Mix and Electronic Health Record Use in Primary Care. *JAMA Netw Open*. Published April 24, 2024.
doi:10.1001/jamanetworkopen.2024.8060

Data
Data available: No
